# The Week's Work

**Published:** 1910-02-19

**Authors:** 


### THE WEEK'S WORK.

### A NEW HOSPITAL JOURNAL.

We have received the first issue of *Het Ziekenhuis* (The Hospital), the official organ of the Netherlands Medical Society for the Improvement of Hospitals in the United Netherlands and the Colonies. This organisation was formed two years ago, already has a large membership, and has done excellent work in promoting a healthy public interest in the affairs of the hospitals in Holland. The new journal is a monthly, admirably printed and edited, and is in charge of a strong editorial committee, at the head of which is Dr. M. A. Mendes de Leon of Amsterdam, whose name is well known on the Continent in connection with institutional medical work. The object of the journal is to give accurate information, plans, and statistics of new and already existing hospitals in Holland and elsewhere, to publish papers by experts on questions of importance in hospital and institutional work, and generally to interest the medical profession and those who are engaged in hospital work in everything that concerns them. The first number opens with an admirable paper by the editor, who takes as his text the often-quoted phrase of Tenon, "Les hôpitaux sont la mesure de la civilisation d'un peuple," and outlines the policy of the paper. Then follows an address by Dr. H. P. Bosscha, President of the Netherlands Association, on the aims of the Society. Dr. Bosscha deals with the vexed question of the justifiability of special hospitals, and it is interesting to note, in view of the recent expression of American opinion on this question, that he has not much to say in favour of special hospitals for infectious diseases. In his opinion it is not desirable to have such special hospitals, since, from a medical point of view, they are unnecessary, while, from the economic point of view, their usefulness is questionable. Nor does he unhesitatingly approve of special hospitals for children, with the exception of infant hospitals. A special ward in a general hospital he considers the ideal, excepting from this, however, chronic ophthalmic, ear, and orthopædic cases. This brings him to the institutions for chronic cases—incurables in a sense—and he lays stress on the desirability of establishing hospitals where such patients may be treated. The paper is most instructive and readable, as it deals also with such questions as the founding of cottage hospitals, of which he is an ardent supporter, the action to be taken to prevent hospital abuse, and the establishment of asylums. Another excellent contribution is the article dealing with the new Boerhaave Clinik at Amsterdam. This is illustrated with well-reproduced photographs and plans, and shows that this newly established private clinic must be ranked as one of the best of its kind in existence. Another article deals with the projected new hospital at Cincinnati. We cordially welcome *Het Ziekenhuis*, which should prove a valuable and instructive journal to those interested in hospital affairs. We congratulate the editorial committee on the success of its first number, and sincerely trust that it may be able to keep up in future issues the high level of excellence which is so conspicuous a characteristic of the January number. The journal is published by the firm of medical publishers, Van Rossen, of Amsterdam, and the yearly subscription is 4 gulden, or 6s. 8d.

### CHARITY ADVERTISING.

Charity advertising requires experience and knowledge not only of what to do and how to do it, but of what to avoid. It is essential to real and continuous success and the best interests of a great charity that the charity should be advertised, and not the individual. Debt should be avoided; every statement in every document issued should be accurate and free from exaggeration; care should be taken to prevent the issue, directly by the charity or indirectly by anybody connected with it, of wild statements, however humorous or telling they may be for newspaper purposes, which tend to irritate givers by exposing their little peculiarities or exhibiting them as geese who may be readily plucked by the wily charitymonger. A clear, telling, accurate statement of the work done and to be done, with guarantees that, when the money is forthcoming but not till then, the objects appealed for will be carried out; the taking of every opportunity to publish widely interesting facts concerning the work and progress of the particular institution; a just recognition of each man's work, and of the results accomplished in the whole, these are a few of the means which, with patience and brains, have not failed in the past and will never fail in the future to secure an adequate revenue for every well-conducted institution under the voluntary system. After all there is such a thing as the business of charity, and in these days, when so many wealthy men give with prodigal liberality, it is essential to success that business methods shall be enforced by the conductors of every great institution if the work it was founded to undertake is to be done to the maximum extent with the maximum efficiency. Fireworks may attract the multitude and please the unthinking. They disgust the real supporters upon whom a great charity has mainly to depend. It is a strange thing to say, but it is the fact, that the large funds now available for distribution by the King's Fund have been obtained by a wise abstention from meretricious appeals and methods for raising money which, though occasionally popular, are deservedly held in contempt by wise and prudent authorities in these matters. The Coronation Gift and the Shopping Day were both proposals which never commended themselves to those who really understand how to raise money for charity. Happily they both disappointed their originators and were soon forgotten. Methods which make most noise in the public press are usually failures, and deservedly failures in the monetary sense. After all, work, steady, intelligent continuous work, is the only way to raise money for charities. Although this way is not popular nowadays, it is one which will have to be continuously followed or the voluntary system of charity, though it at present produces some fifteen millions a year, must gradually languish and disappear.

### THE CANADIAN HOSPITAL ASSOCIATION.

The report of the third annual meeting of the Canadian Hospital Association, held in April last, has just been published and is an interesting and instructive little pamphlet, which contains many hints to those who are engaged in hospital and institutional work. In it Mr. Edward Stevens, who has planned various American hospitals, and is at present engaged upon designs for several cottage hospitals, gives details of his work in a little paper on "Some points in the architecture of small hospitals," which is illustrated by plans. He prefers a 30- to 40-bed establishment built on a system which admits of expansion to 80 beds, should need for enlargement arise. Dealing with infectious cases, he strongly urges the employment of one main isolation ward, and insists that such cases are "better managed by the general hospital than in an independent institution." He is against providing a separate ward for each variety of infectious disease, and points out that in the Pasteur Hospital in Paris, which in many points may be looked upon as an ideal isolation hospital, all cases are treated in one building. The obvious objection to this method is, of course, the danger of cross-infection; but he shows that at the Pasteur Hospital during five years the cases of cross-infection recorded have not attained the total of two in a thousand. This, as he justly observes, "is more than can be said of many hospitals where each disease is segregated from the others." He lays stress on the isolation balcony, which should be provided with large glass screen walls, through which visitors and friends of patients can communicate with the latter from the outside without risk of infection. Smaller points in his paper are the excellent descriptions given of modern operating theatres, of the children's ward, and of the maternity building. It is interesting to note that Mr. Stevens, after experimenting with all types of floors, announces his preference for the jointless linoleum floor cemented to a concrete base. There are several nursing articles, one of which deals with the inadvisability of training a nurse for one year in a small hospital with the idea of final instruction in a large general hospital. Dr. Boyce, Superintendent of the Kingston General Hospital, contributes a paper on the evolution of surgical technique, and Dr. Campbell Meyers, one on the establishment of special neuropathic wards in a general hospital. Such wards have been established at the Toronto Hospital and have been found quite satisfactory. The concluding paper is by Miss Robinson, and gives a summary of the help that a properly-organised Women's Hospital Aid Association can give an institution not only by subscribing funds for special objects, but by interesting others in the work of the charity, in maintaining special beds, and in helping to provide clothing and comforts for the patients.

### THE DRUG MARKET.

The Cod Liver Oil market is being watched with interest, and prices show a tendency to advance. Later reports from Norway concerning the cod-fishing are not so favourable, and the weather at the fishing grounds has been stormy. It is also evident that the livers are leaner than last year, and the oil yield of four of this season's livers is only about equal to three of last season's. The catch up to the present has been good, however, and there are no indications at present that the total yield of oil will be materially below that of an average year. The high value of ipecacuanha is maintained, and extreme prices are still asked for Damiana leaves. On the other hand, Buchu leaves show indications of receding in value, if only to a slight extent. The price of codeine has been reduced by about one shilling per ounce.

